# Predictors of mass psychogenic illness in a junior secondary school in rural Botswana: A case control study

**DOI:** 10.4102/sajpsychiatry.v28i0.1671

**Published:** 2022-05-30

**Authors:** Keatlaretse Siamisang, Thabo Phologolo, Terrence Mukuhwa, Nathaniel Schafrick, Bonolo Mhaladi, Boang Phuthego, Monica Mmati, Tiny Masupe

**Affiliations:** 1 Department of Family Medicine and Public Health, Faculty of Medicine, University of Botswana, Gaborone, Botswana; 2 Department of Health Services Management, Ministry of Health and Wellness, Gaborone, Botswana; 3 Department of Family Medicine and Public Health, Faculty of Medicine, University of Vermont, Vermont, United States of America

**Keywords:** mass psychogenic illness, predictors, Botswana, case control, school, mass hysteria

## Abstract

**Background:**

In March 2019, students at Lempu Secondary School in Kweneng District, Botswana displayed symptoms including headache, abnormal leg movements and difficulty walking. Within days, 133 students were admitted to Scottish Livingstone Hospital where mass psychogenic illness (MPI) was diagnosed.

**Aim:**

To identify predictors of this illness.

**Setting:**

Kweneng West District, Botswana.

**Methods:**

This was a case control study using interviewer-administered questionnaires. Cases were students who displayed MPI symptoms from the 2nd of March to the time of the interviews or who were admitted with MPI diagnosis. Analysis was restricted to female students. Logistic regression was used to generate odds ratios. A *p* value of < 0.05 was considered to demonstrate significant association between variables.

**Results:**

Interviews were conducted with 142 cases and 202 controls. The median age was 15 years. Most of the cases (95.8%) were boarding girls. Residence in school campus (AOR 13.2), history of evaluation by psychologist and/or social worker (AOR 2.6), history of traumatic events (AOR 1.8), contact with sick peers (AOR 2.3) and contact with spiritual healer (AOR 2.0) were independent predictors of MPI. Additionally, perception of adequate security in the dormitories (AOR 0.3) and perception of poor lighting (AOR 6.8) were significant predictors of MPI amongst boarding girls.

**Conclusion:**

The outbreak in Lempu Community Junior Secondary School (CJSS) was typical of mass psychogenic illness affecting mainly boarding girls and was associated with psychological and environmental risk factors. Changing the boarding environment and continuous psychological support are key to preventing future outbreaks. Interventions should also target the identified risk factors.

## Introduction

Mass psychogenic illness (MPI), commonly referred to as mass hysteria, is the rapid spread of an illness amongst a cohesive group that unconsciously manifests with physical signs and symptoms which originate from a nervous system disturbance involving excitation, loss or alteration of function.^1,2^ Whilst the disease symptoms may suggest a physical illness, physical examination and laboratory investigations do not identify any organic cause.^3,4,5^ Mass psychogenic illness is widely believed to belong to a group of psychiatric disorders known as conversion disorders.^6,7^ Patients with these disorders tend to present with physical symptoms but with no objective motor abnormalities in the neurological examination. Furthermore, the abnormal symptoms are generally not consistent with any known neurological disease.^6,8^ The term ‘conversion’ refers to manifestation of physical symptoms in place of suppressed emotions or ideas.^9^ Unlike malingering, symptoms of conversion disorders are not under the voluntary control of the patients.^10^ There is often a perception of threat associated with a disproportionate reaction. Mass psychogenic illness most commonly affects people who live in institutions or in groups. The most common setting for epidemic MPI is schools, particularly boarding institutions.^1^ Other commonly affected settings include factories, villages, prisons and religious institutions.^11^

The hallmark of MPI is that cases spread rapidly through visual, sound and physical contact, and it predominantly affects the female gender.^1^ When these hallmark presentations are associated with presence of extraordinary anxiety and spread down age scale beginning with older or higher status people, a presumptive diagnosis of MPI can be made.^12^ In making a diagnosis of MPI, potentially catastrophic organic alternative diagnoses like infections and poisoning must be ruled out. Indeed, MPI and organic outbreak are not mutually exclusive. A small outbreak of a physical illness may cause great anxiety in a community leading to rapid development of MPI.

Mass anxiety hysteria and mass motor hysteria are the two main forms of MPI described in the literature.^1,13,14^ Mass anxiety hysteria is most often seen in schools, especially boarding institutions. Often there is sudden stress in a group of school children.^13^ This form generally spreads rapidly through a close-knit group via sight and physical contact with affected individuals.^1^ The most common symptoms are headache, dizziness, hyperventilation and fainting. Mass anxiety hysteria has a good prognosis and separating the affected group is an effective treatment.^14^ Mass motor hysteria, on the other hand, is less common and tends to affect all age groups. The spread is more gradual and epidemics tend to be prolonged.^1^ Typically, affected individuals have longstanding anxiety.^13^ The most common symptoms are twitching, difficulty walking and inappropriate laughter. Removal of the stressor is a key component of the treatment. However, symptoms may persist after withdrawal of the stressor.^13,14^

Students at Lempu Secondary School in Kweneng West District, Botswana suddenly displayed unusual symptoms in March 2019. The first case was that of a previously well female boarding student on Saturday 02 March 2019. She presented with sudden onset of difficulty maintaining normal standing posture and abnormal leg movements whilst standing, similar to dancing, with no other symptoms. She had intact mentation and no cause of her symptoms was identified after review at the local clinic. By Monday 04 March 2019, three more female students were assessed at the local clinic with similar presentation. As cases increased, students presented with variable complaints which ranged from hallucinations, numbness of the lower extremities as well as visual impairment and perception of blindness. The illness spread rapidly through sight and contact with affected individuals. On Wednesday 06 March 2019, a decision was made to refer the symptomatic students to Scottish Livingstone Hospital, a district hospital about 130 km from the village. After the decision, the number of affected students rose sharply and 120 students were sent for hospital admission. The school was temporarily closed on 07 March 2019 and reopened on 18 March 2019. The outbreak was ongoing at the time of this study.

To our knowledge, this is the first report of an MPI investigation in Botswana. There is therefore very little knowledge of this condition in the country. Furthermore, case control studies on this condition are rare in the general literature. Most of the studies have been descriptive and have not investigated the individual factors associated with development of the illness.^1,15,16^ To this end, little is known about the risk factors of MPI in our setting. This study therefore significantly adds to the growing global body of evidence on this poorly understood phenomenon. The aim of this study was to investigate the MPI outbreak and to identify the predictors of the illness at Lempu Junior Secondary School.

## Methods

### Study setting

The study was conducted in Lempu Junior Secondary School in Salajwe, a rural village of about 3,500 inhabitants located about 190 km from the capital city, Gaborone. The boarding school caters for students from Salajwe and nearby villages of Khudumelapye, Malwelwe, Kaudwane, Monwane and Sorilatholo. In the Botswana public education system, the first 7 years after preschool are spent in a primary school. Learners then spend 3 years in a junior secondary school. These years are formally called Form 1, Form 2 and Form 3.

### Study design

This was a case control study done as part of an outbreak investigation of MPI in Lempu School. The cases and controls were to be matched for sex but because only one male case was interviewed, analysis was restricted to female students. Multiple exposures were examined amongst the cases and controls.

### Definition of cases and controls

Cases were defined as any student attending Lempu Community Junior Secondary School who developed one or more of the following symptoms: abnormal leg movements, difficulty walking, hallucinations, delusions, perception of visual impairment or loss of sight from Saturday 02 March 2019 until the day of the interview or who had been admitted to Scottish Livingstone Hospital with a diagnosis of MPI or hysteria in the same period. Controls were students at the school who did not meet the criteria for the case definition.

### Selection of participants

Sampling of cases was exhaustive. All cases that consented were enrolled into the study. The remaining female students were interviewed as controls. Male controls were to be selected by simple random sampling, using the school register as a sampling frame. However, the analysis was restricted to female students because there was only one male case.

### Data collection

Data were collected between 06 May 2019 and 17 May 2019. A structured, interviewer-administered questionnaire was used to collect information on baseline characteristics of students, possible exposures and predisposing factors for MPI as well as perception of the school environment. Questions included information on demographics, school performance and engagement, as well as medical, psychiatric and social history. In addition, we administered the Patient Health Questionnaire-2 (PHQ-2) and the General Anxiety Disorder 7-item scale (GAD-7) to screen for depression and GAD, respectively. These tools have proven validity and efficiency in screening for these disorders.^17,18,19,20,21^ The PHQ-2 includes the first two questions of the more comprehensive PHQ-9 and enquires about the frequency of anhedonia and depressed mood in the preceding 2 weeks.^22^ The GAD-7 is a 7-item questionnaire about anxiety symptoms experienced in the preceding 2 weeks.^23^

### Analysis

EpiInfo^TM^ software version 7.2 was used for data capturing and analysis. Categorical data was summarised with frequencies and percentages whilst numeric data was summarised with means, standard deviation, medians and interquartile ranges. Pearson’s chi square test and Fisher’s exact test were used to compare different categorical data. A *p*-value of <0.05 was considered to demonstrate significant association between variables. Bivariate and multiple logistic regression were used to determine factors associated with development of the illness. A *p* value of 0.25 was used as a cut-off point to recruit variables from the bivariate model into the multiple regression model. A *p* value of < 0.05 was considered significant in the multivariate model. The results of logistic regression were presented in the form of odds ratios.

### Ethical considerations

The study involved a vulnerable group and had to address a range of ethical issues. As part of the District Health Management Team (DHMT) response to the outbreak, the study was expected to benefit participants directly. This was achieved by targeted interventions, as more information was made available. Consultative meetings were held with parents, caregivers and/or guardians of the students and consent sought for participation. Clear information about the study was given to potential study participants, and as they were below the age of 18 years, assent was sought. Participants were informed that they could withdraw at any time with no repercussions. Data collection was done in private and information about all participants was kept private and confidential. Identifying information was removed from the participants’ data. Psychiatric nurses were available on site to assess students who scored high on PHQ-2, GAD-7 or had suicidal ideation, whilst a team of psychiatrists was available for referral for participants who required immediate and urgent interventions. Ethics approval was obtained from the first author’s Institutional Review Board and from the Botswana Ministry of Health and Wellness ethics committee.

## Results

A total of 344 students were interviewed, of which 142 female students met the case definition of MPI. The rest (202) of the female students were unaffected and were interviewed as controls, resulting in a case control ratio of 1:1.4. Table 1 shows the baseline characteristics of the study participants. The age range was 12–18 for both cases and controls, and the median age was 15 years. A total of 136 (95.8%) cases and 136 (67.3%) controls were boarding students (*p* < 0.001). Most of the affected students were from the villages of Khudumelapye (32.4%), Sorilatholo (21.8%), Malwelwe (21.8%), Kaudwane (14.1%) and Monwane (6.3%). Only 2.1% of the participants were local residents of Salajwe village. Form 1 students accounted for 38.7% of cases whilst form 2 and 3 students accounted for 35.9% and 24.6% of cases, respectively.

**TABLE 1:** Baseline characteristics of cases and controls.

Variable	Characteristics	Cases (*n* = 142)					Controls (*n* = 202)					*p*
		*n*	%	Median	IQR	Range	*n*	%	Median	IQR	Range	
Age	-	-	-	15	14–15	12–18	-	-	15	14–16	12–18	-
Home Village	Kaudwane	20	14.1	-	-	-	29	14.4	-	-	-	-
	Khudumelapye	46	32.4	-	-	-	65	32.2	-	-	-	-
	Malwelwe	31	21.8	-	-	-	23	11.4	-	-	-	-
	Monwane	9	6.3	-	-	-	6	3.0	-	-	-	-
	Sorilatholo	31	21.8	-	-	-	18	8.9	-	-	-	-
	Salajwe	3	2.1	-	-	-	58	28.7	-	-	-	-
	Not documented	2	1.5	-	-	-	3	1.5	-	-	-	-
Year of study	Form 1	55	38.7	-	-	-	79	39.1	-	-	-	-
	Form 2	51	35.9	-	-	-	69	34.2	-	-	-	-
	Form 3	35	24.6	-	-	-	52	25.7	-	-	-	-
Boarding	-	136	95.8	-	-	-	136	67.3	-	-	-	< 0.001*
History of bullying	-	34	23.9	-	-	-	57	28.2	-	-	-	0.19
Grade C or better (Last term)	-	83	58.4	-	-	-	112	55.4	-	-	-	0.24
Participation in sports	-	68	47.9	-	-	-	87	43.0	-	-	-	0.18
Participation in athletics	-	36	25.4	-	-	-	47	23.3	-	-	-	0.33
Participation in clubs	-	37	26.1	-	-	-	38	18.8	-	-	-	0.06
Chronic medical illness	-	13	9.1	-	-	-	15	7.4	-	-	-	0.29
History of mental illness	-	3	2.1	-	-	-	2	1.0	-	-	-	0.22
Use of chronic medications	-	8	5.6	-	-	-	7	3.4	-	-	-	0.17
Family size	-	-	-	6	4–7	-	-	-	6	4–8	-	
Perception of good academic support at home	-	16	11.3	-	-	-	52	25.7	-	-	-	0.18
Family use of social services	-	61	43.0	-	-	-	89	44.1	-	-	-	0.09
Christian religion affiliation	-	132	93.0	-	-	-	188	93.0	-	-	-	0.46

IQR, interquartile range.

* , *p* < 0.05.

Students’ perceptions of their school environment are presented in Table 2. The majority of cases and controls reported adequate academic and social support. They also reported good relationships with staff. Most of the cases (82.4%) and controls (74.3%) reported a good relationship with their teachers and school management. There were significant differences in the perception of cleanliness in the classrooms and ablution facilities, with 52.1% of cases and 68.8% of controls reporting that their classrooms were clean (*p* = 0.002) and only 7.0% of cases and 14.9% of controls reporting clean ablution facilities (*p* = 0.01). Similarly, the majority of study participants believed that the overall school cleanliness was poor. Only 8.5% of cases and 8.9% of controls believed that the school was clean (*p* = 1.00). Amongst boarding students, there were significant differences between cases and controls in the perception of cleanliness, security and good lighting in the dormitories. A smaller proportion of cases (40.1%) believed that the dormitories were adequately lit at night compared to controls (53.0%) (*p* < 0.001). Similarly, cases (31.0%) were less likely than controls (38.6%) to believe that the dormitories were secure (*p* < 0.001). A higher proportion of controls thought that the facilities were not clean with 45.8% of cases and 33.8% of controls reporting good cleanliness (*p* = 0.03). Only 50% of cases and 52% of controls believed that the school management response to the outbreak was good (*p* = 0.80). Significantly higher proportions of participants (cases: 85.9%, controls 82.1 % *p* = 0.06) approved of the government’s response to the outbreak.

**TABLE 2:** Students’ perceptions of the school environment.

Variable	Cases (*n* = 142)		Controls (*n* = 202)		*p*
	*n*	%	*n*	%	
Perception of good academic support	115	81.0	155	76.7	0.36
Perception of adequate social support	89	62.7	127	62.9	1.00
Perception of good relationship with teachers	117	82.4	150	74.3	0.09
Participation in wellness activities	57	40.1	63	31.2	0.11
Perception of cleanliness in the classroom	74	52.1	139	68.8	0.002*
Perception of cleanliness in the ablutions	10	7.0	30	14.9	0.01*
Perception of overall school cleanliness	12	8.5	18	8.9	1.00
Perception of adequate security during school hours	31	21.8	60	29.7	0.13
Perception of food freshness	45	31.7	64	31.7	1.00
Perception of cleanliness in the dormitories	65	45.8	46	33.8	0.03
Perception of security in the dormitories	44	31.0	78	38.6	< 0.001*
Perception of good lighting in the dormitories	57	40.1	107	53.0	< 0.001*
Perception of good response to the outbreak by school	71	50.0	105	52.0	0.80
Perception of good response to the outbreak by the government	122	85.9	166	82.1	0.06

* , *p* < 0.05.

The background medical history was generally not significantly different between the cases and controls (Table 3). However, 38.3% of cases had travelled in the month before the outbreak compared to 26.4% of controls (*p* = 0.03). Additionally, cases reported higher rates of history of traumatic events (45.4% vs 30.0%, *p* = 0.003), history of evaluation by psychologist or social worker (48.2% vs 25.0%, *p* < 0.001) and contact with spiritual healers (70.2% vs 52.0%, *p* < 0.001). None of the participants reported smoking or use of illicit drugs whilst 12.7% of cases and 11.4% of controls reported use of alcohol (*p* = 0.86). Suicidal ideation was reported in 7.0% of cases and 8.5% of controls (*p* = 0.77), whilst 2.1% of cases and 3.0% of controls reported past history of suicide attempts (*p* = 0.87). In depression screening, the PHQ-2 score was abnormal (>2) in 4.9% of cases and 1.4% of controls (*p* = 0.12). Similarly, 5.6% of cases and 4.0% of controls had an abnormal GAD-7 score of >7 (*p* = 0.64).

**TABLE 3:** Participants’ medical and psychological history.

Variable	Cases		Controls		*p*
	*n*	%	*n*	%	
Chronic medical condition	13	9.2	15	7.5	0.72
Long term medications	8	5.7	7	3.5	0.48
New medications before outbreak	32	23.0	35	17.5	0.26
History of alcohol use	18	12.7	23	11.4	0.86
Travel before outbreak	54	38.3	52	26.4	0.03*
Current mental illness	3	2.1	2	1.0	0.70
Previous mental illness	2	1.4	1	0.5	0.76
History of suicidal attempts	3	2.1	6	3.0	0.87
History of suicidal ideation	10	7.0	17	8.5	0.77
Current suicidal ideation	5	3.5	5	2.5	0.82
History of traumatic events	59	45.4	55	30.0	0.003*
History of evaluation by psychologist or social worker	68	48.2	49	25.0	< 0.001*
Contact with traditional healers	13	9.2	31	15.8	0.10
Contact with spiritual healers	99	70.2	104	52.0	0.001*
PHQ score > 2	7	4.9	3	1.4	0.12
GAD-7 score > 7	8	5.6	8	4.0	0.64

PHQ, Patient Health Questionnaire; GAD-7, General Anxiety Disorder 7-item scale.

* , *p* < 0.05.

The predictors of MPI are presented in Table 4. Student boarding (adjusted odds ratio [AOR] 13.2, 95% CI 4.7–37.0), evaluation by psychologist or social worker (AOR 2.6, 95% CI 1.5–4.5), history of traumatic events (AOR 1.8, 95% CI 1.0–3.1), history of contact with sick peers (AOR 2.3, 95% CI 1.2–4.6) and history of contact with spiritual healers (AOR 2.0, 95% CI 1.1–3.3) were statistically and significantly associated with MPI in the adjusted model. When limiting the analysis to female boarding students (Table 5), history of evaluation by psychologist or social worker remained statistically and significantly associated with the outcome (AOR 2.6, 95% CI 1.3–5.5). Additionally, female boarding students who thought their dormitory was secure had significantly lower odds of MPI (AOR = 0.3, 95% CI 0.2–0.7). Furthermore, perception of poor lighting in the dormitory was associated with higher odds of MPI (AOR 6.8, 95% CI 0.2–0.7).

**TABLE 4:** Association between mass psychogenic illness and independent variables (multivariate model).

Variable	Adjusted odds ratio	Lower 95% CI	Upper 95% CI	*p*
Boarding (yes or no)	13.2	4.7	37.0	< 0.001*
Perception of good relationship with teachers (good or bad)	1.0	0.3	3.4	0.98
Perception of security during school hours (good or bad)	1.5	0.81	2.8	0.20
Use of prescription medications (yes or no)	1.59	0.81	3.1	0.18
Recent acute illness (yes or no)	1.1	0.56	2.1	0.82
Recent travel history (yes or no)	1.6	0.87	2.9	0.13
Anxiety screen cut-off (GAD7 < 7 or GAD7 > 7)	4.0	0.44	37.4	0.22
Depression screen cut-off (PHQ > 2 or PHQ < 2 or =2)	1.3	0.30	5.9	0.71
History of traumatic events (yes or no)	1.8	1.0	3.1	0.05*
Evaluation by psychologist or social worker (yes or no)	2.6	1.5	4.5	< 0.001*
History of contact with spiritual healers (yes or no)	2.0	1.1	3.3	0.02*
History of contact with sick peers (yes or no)	2.3	1.2	4.6	0.02*

PHQ, Patient Health Questionnaire; GAD-7, General Anxiety Disorder-7 item scale.

* , *p* < 0.05.

**TABLE 5:** Significant predictors of mass psychogenic illness amongst boarding girls (multivariate model).

Variable	Odds ratio	95% CI	*p*
Ever evaluated by psychologist or social worker (yes or no)	2.6	1.3–5.5	0.01*
Lighting in the dormitory (not lit or lit)	6.8	3.1–14.3	< 0.001*
Security in the dormitory (secure or not secure)	0.3	0.2–0.7	0.002*

CI, confidence interval.

* , *p* < 0.05.

## Discussion

The outbreak in Lempu Junior Secondary School was typical of MPI, which often affects otherwise well individuals who tend to attribute their illness to an external factor usually in the context of significant long-term stress.^16^ Many of the students in Lempu were not happy with their school environment, particularly the cleanliness and security, which could be a cause of long-term stress. Female students accounted for 98.5% of affected individuals. This is consistent with MPI in previously reported outbreaks.^5,16,24,25^ In a study on MPI in Korea following H1N1 vaccination, females were disproportionately represented amongst victims.^26^ Many of them required prolonged hospitalisation of more than 7 days. In another study, Tarafder et al. reported an episode of MPI amongst girls in a similar age group in a school setting. However, the episode was short with most of the cases hospitalised for less than 12 h.^2^ The MPI episode in Lempu was much longer and was ongoing at the time of the interviews.

The cases from this study were from the villages of Monwane, Khudumelapye, Kaudwane, Sorilatholo and Malwelwe. Only three cases were from Salajwe. This was because most of the students from Salajwe are day-scholars and the illness affected mainly boarding students (95.8%). The predominance of boarding students is consistent with MPI, which typically affects school children in boarding institutions.^20^ Students at all levels of study were affected. This is consistent with other school-based outbreaks which did not show any association between year of study and development of the illness.^15,16^

The majority of cases and controls reported adequate academic and social support and good relationships with teachers. Notably, cases were not more likely to report poor support or relationship with teachers and management than controls. Rather, a higher proportion of cases reported good relationships than controls; however, this difference was not statistically significant. These factors were therefore not found to be contributing to the MPI outbreak in Lempu. Both cases and controls were mostly not happy with the overall school cleanliness. Only about 9% reported that the school was clean. The cases were significantly less likely to report clean classroom and ablution facilities. This not only demonstrates that the vast majority of students reported their school is not clean, but also implies a positive association between perception of cleanliness and development of MPI.

There were significant differences in the perceptions of cleanliness, security and good lighting in the dormitories between the cases and controls. A higher proportion of cases believed the dormitories were not well lit or secure. This could be the source of significant stress that ultimately triggered the MPI outbreak in the school. Interestingly, a higher proportion of controls thought the facilities were not clean. The majority of cases and controls believed that food served in the school was not fresh. Food provided at a school has been at the centre of MPI outbreak previously. Haque et al. described an outbreak in Bangladesh following ingestion of biscuits provided at a primary school.^15^ However, the cases were much younger and there was a rapid recovery of symptoms. Still in Bangladesh, MPI outbreak resulted from consumption of cake at a girls’ high school.^2^ Qualitative studies on perceptions and experiences of the MPI episode are needed to explore these factors at Lempu Junior Secondary School.

Participants were interviewed about their current and past medical history to look for any clues of an organic illness that could have been linked to the outbreak. Specific questions were directed towards risk factors of infection, particularly travel and contact history. An infectious outbreak is a major differential for MPI and must be ruled out.^27^ Travel history was significantly more frequent amongst the cases than the controls. This, however, could be accounted for as most of the cases were boarding students from other villages and therefore required travel to visit their families during vacation time. Indeed, history of travel was not a predictor of the illness in the multivariate regression model (Table 4). The difference in travel was therefore not considered to be a risk factor for the outbreak. Differences in contact history could be explained by the fact that MPI is spread through physical and visual contact.^1,5,16^ Medical history and medications use was not different between the cases and controls. Cases were not more likely to use alcohol than controls and none of the participants reported history of smoking or use of illicit drugs. This may be because all the study participants were girls. In secondary school settings, smoking has been reported to be more common amongst male students.^22^

Five factors had an independent association with development of the illness. Boarding students were 13.2 times more likely to be affected than day students after controlling for multiple potential confounders. This further supports an environmental trigger in the school premises or boarding facilities and is also consistent with MPI. This illness is very common in boarding schools with 50% of reported episodes being in this setting.^1^ Past history of traumatic events increased the odds of the illness by 77%. Previous traumatic events or loss have been associated with MPI in previous studies. Small et al. reported an association between hospitalisation for MPI and parental divorce and death within the family.^28^ History of psychological trauma is an established risk factor for psychogenic illness.^7^ Furthermore, study participants with history of trauma may have underlying undiagnosed psychiatric illnesses that may predispose them to MPI. Students who reported prior evaluation by psychologist or social worker were 2.6 times more likely to be affected than their other colleagues. This may be because they were more likely to have underlying psychosocial issues that would predispose them to the illness. Furthermore, these students may be less likely to cope in a stressful school environment than their colleagues. This association was also reported in an Ethiopian case control study in which students who reported psychological stress were 2.6 times more likely to be affected than their peers.^16^

History of use of spiritual healer services increased the odds of the illness by almost 100%. This could mean certain religious or spiritual beliefs may be associated with the illness. Indeed, spiritual beliefs are often involved in development of MPI episodes in developing countries. Often these episodes are attributed to evil spirits, demons or spirits of ancestors.^25^ Lake et al. found a positive association between perception of evil or demonic force and development of psychogenic illness.^16^ A qualitative study would help explore the spiritual beliefs and experiences associated with MPI in Lempu School. Lake et al. also noted that students who reported seeing affected peers were 2.9 times more likely to be affected themselves than those who did not. A similar association was found in our study in Lempu Junior School. Students who reported physical contact with sick peers were 2.3 times more likely to develop symptoms of the illness than those who did not. This has been consistently reported in the literature. Mass psychogenic illness is mainly spread through physical contact or seeing affected individuals.^1,5,16^ There was no association between development of the illness and academic performance. This is consistent with a case control study in Bangladesh which found no association between MPI and academic performance.^25^

Surprisingly, there was no association between the depression and anxiety scores and the development of MPI in our study. This is in contrast to a multicentre school-based survey aimed at determining adolescents’ hysterical tendencies in China.^29^ The authors reported a significant association between Hospital Anxiety and Depression Scale (HADS) and hysterical tendencies. However, in our study we used PHQ and Generalized Anxiety Depression Score (GAD). There is only moderate agreement between HADS and PHQ in detecting depression.^30^ This may explain the different findings. Furthermore, the Chinese study measured a different outcome of hysterical tendencies and not actual development of MPI. Additionally, being witness to the number of cases seen at the school may have increased the levels of anxiety and depression amongst controls. Amongst boarding girls, perception of poor lighting and security in the facilities were both associated with development of the outcome. Boarding girls who thought their dormitories were not well lit and those who were not happy with the security in the school were more likely to have MPI. This further indicates that stress related to the boarding environment could be a trigger for the MPI episode.

## Limitations

This study had some limitations. Interviews were conducted during the outbreak when some students still had symptoms. Persistent anxiety, uncertainty and confusion about the nature of the illness could have affected the participants’ ability to answer the questions accurately. There was also interaction of participants, meaning discussions amongst students could not be ruled out. Like all case control studies, we could not rule out misclassification due to recall bias or interviewer bias. However, a structured questionnaire was used and interviewers were trained on appropriate uniform questioning of participants and interviewing skills. Finally, suggestibility was not measured in this study. Although suggestibility most often affects young children, it cannot be ruled out in adolescent interviews and may affect their responses leading to bias.^31^

## Conclusion

The MPI outbreak in Lempu CJSS affected mainly boarding girls and was associated with history of social worker or psychology evaluation, contact with sick peers, use of spiritual healer services, perceptions of poor lighting and safety at the dormitories and reported history of traumatic events. In this outbreak, changing the school environment was the key aspect of outbreak management, particularly improving safety and security at the boarding facilities and providing ongoing comprehensive psychosocial evaluation and support. This intervention must be holistic and should include parents, guardians, teachers and community leaders. Student assessments should be made at enrollment to identify psychologically vulnerable students who would require more psychological support.

Further qualitative studies are needed to assess the experiences and perceptions of students, school management, parents and the community during and after school-based MPI outbreaks. This will give a more in-depth understanding of the phenomenon and may raise new issues that are not captured in the quantitative study. A different perspective would also allow triangulation with the results of the current study.

## References

[1] Ayehu M, Endriyas M, Mekonnen E, Shiferaw M, Misganaw T. Chronic mass psychogenic illness among women in Derashe Woreda, Segen Area People Zone, southern Ethiopia : A community based cross – Sectional study. Int J Ment Health Syst. 2018;12:31. 10.1186/s13033-018-0207-1 29930699 PMC5992694

[2] Faruq I, Islam MT, Tarafder BK, et al. Mass psychogenic illness: Demography and symptom profile of an episode. Psychiatry J. 2016;2016:1–5. 10.1155/2016/2810143 PMC4884863 27294104

[3] Magnavita N. Industrial mass psychogenic illness: The unfashionable diagnosis. Br J Med Psychol. 2000;73(3):371–375. 10.1348/000711200160570 11003376

[4] Schaffner W, Craig AS, Gunter EW, et al. Mass psychogenic illness attributed to toxic exposure at a high school. N Engl J Med. 2002;342(2):96–100. 10.1056/NEJM200001133420206 10631279

[5] Keshishian C. Mass psychogenic illness and how to respond to incidents. The magazine of the Health Protection Agency [serial online]. 2009 [cited 2020 Oct 1]; (13):22–23. Available from: https://www.apothecaries.org/wp-content/uploads/2019/06/Mass-psychogenic-illness-and-how-to-respond.pdf

[6] Kaur J, Garnawat D, Ghimiray D, Sachdev M. Conversion disorder and physical therapy. Delhi Psychiatry J [serial online]. 2012 [cited 2020 Oct 1]; 15(2):394–397. Available from: http://medind.nic.in/daa/t12/i2/daat12i2p394.pdf

[7] Maldonado J, Spiegel D. Conversion disorder. Review of psychiatry [homepage on the Internet]. 2017 [cited 2020 Oct 1]. Available from: https://www.researchgate.net/publication/285811711_Conversion_disorder

[8] Rofé Y, Rofé Y. Conversion disorder: A review through the prism of the rational-choice theory of neurosis. Eur J Psychol. 2013;9(4):832–868. 10.5964/ejop.v9i4.621

[9] Ali S, Jabeen S, Pate RJ, Shahid M, Chinala S, Nathani S, et al. Innov Clin Neurosci. 2015 [cited 2020 Oct 1]; 12(5):27–33. Available from: https://www.ncbi.nlm.nih.gov/pmc/articles/PMC4479361/PMC4479361 26155375

[10] Kendall EA, Zaman RU, Naved RT, Arman S, Rahman MW, Kadir MA, et al. Medically unexplained illness and the diagnosis of hysterical conversion reaction (HCR) in women’s medicine wards of Bangladeshi hospitals: A record review and qualitative study. BMC Womens Health. 2012;12(1):1–10. 10.1186/1472-6874-12-38 23088583 PMC3534409

[11] Balaratnasingam S, Janca A. Mass hysteria revisited. Curr Opin Psychiatr. 2006;19(2):171–174. 10.1097/01.yco.0000214343.59872.7a 16612198

[12] Weir E. Mass sociogenic illness. CMAJ. 2005;172(1):36. 10.1503/cmaj.045027 15632400 PMC543940

[13] Bartholomew RE, Wessely S, Rubin GJ. Mass psychogenic illness and the social network: Is it changing the pattern of outbreaks? J Roy Soc Med. 2012;105(12): 509–512. 10.1258/jrsm.2012.120053 23288084 PMC3536509

[14] Wessely S. Mass hysteria: Two syndromes? Psychol Med. 1987;17(1):109–120. 10.1017/S0033291700013027 3575566

[15] Haque F, Kundu SK, Islam MS, et al. Outbreak of mass sociogenic illness in a school feeding program in Northwest Bangladesh, 2010. PLoS One. 2013;8(11):1–8. 10.1371/journal.pone.0080420 PMC3828262 24244685

[16] Lake M, Erku M, Hailu H, et al. Hysteria outbreak investigation in Kombolcha town among school girls, northwest Ethiopia, january, 2013. Sci J Public Health. 2016;4(1):37–42. 10.11648/j.sjph.20160401.15

[17] Spitzer RL, Kroenke K, Williams JBW, et al. A brief measure for assessing generalized anxiety disorder: The GAD-7. Arch Intern Med. 2006;166(10): 1092–1097. 10.1001/archinte.166.10.1092 16717171

[18] Dadfar M, Lester D. Psychometric Characteristics of Patient Health Questionnaire-2 (PHQ-2) in Iranian psychiatric outpatients. Austin J Psychiatru Behav Sci [serial online]. 2017 [cited 2020 Oct 1]; 4(1):1–6. Available from: https://austinpublishinggroup.com/psychiatry-behavioral-sciences/fulltext/ajpbs-v4-id1059.pdf

[19] Richardson LP, Rockhill C, Russo JE, et al. Evaluation of the PHQ-2 as a brief screen for detecting major depression among adolescents. Pediatrics. 2010;125(5): 1097–1103. 10.1542/peds.2009-2712 PMC3100798 20368315

[20] Seo JG, Park SP. Validation of the Patient Health Questionnaire-9 (PHQ-9) and PHQ-2 in patients with migraine. J Headache Pain. 2015;16(1):65. 10.1186/s10194-015-0552-2 26174509 PMC4501946

[21] Lowe B, Decker O, Muller S, et al. Validation and standardization of the Generalized Anxiety Disorder screener (GAD-7) in the general population. Med Care. 2009;46(3):266–274. 10.1097/MLR.0b013e318160d093 18388841

[22] Arrieta J, Aguerrebere M, Raviola G, et al. Validity and utility of the Patient Health Questionnaire (PHQ)-2 and PHQ-9 for screening and diagnosis of depression in rural Chiapas, Mexico: A cross-sectional study. J Clin Psychol. 2017;73(9): 1076–1090. 10.1002/jclp.22390 28195649 PMC5573982

[23] Williams N. The GAD-7 questionnaire. Occup Med [serial online]. 2014 [cited 2020 Oct 1]; 64(3):224. Available from: https://www.researchgate.net/publication/7064924_A_Brief_Measure_for_Assessing_Generalized_Anxiety_Disorder_The_GAD-7/link/0912f50a8d0815d27a000000/download

[24] Govender I. Mass hysteria among South African primary school learners in Kwa-Dukuza, Kwazulu-Natal. S Afr Fam Pract. 2010;52(4):318–321. 10.1080/20786204.2010.10873998

[25] Mamun A, Maruf M, Bhowmik A, et al. Mass psychogenic illness: Comparison on selected variables between cases and non-cases. Bangladesh J Psychiatry. 2018;30(1):14–19. 10.3329/bjpsy.v30i1.37857

[26] Yang T, Kim H, Lee Y, et al. Psychogenic illness following vaccination: Exploratory study of mass vaccination against pandemic influenza A (H1N1) in 2009 in South Korea. Clin Exp Vaccine Res. 2017;6(1):31–37. 10.7774/cevr.2017.6.1.31 28168171 PMC5292354

[27] Goldstein DM, Hall K. Mass hysteria in Le Roy, New York: How brain experts materialized truth and outscienced environmental inquiry. Am Ethnol. 2015;42(4):640–657. 10.1111/amet.12161

[28] Small G, Nicholi A. Mass hysteria among schoolchildren. Early loss as a predisposing factor. Arch Gen Psychiatry. 1982;39(6):721–724. 10.1001/archpsyc.1982.04290060065013 7092505

[29] Cheng Q, Xie L, Hu Y, et al. Gender differences in the prevalence and impact factors of hysterical tendencies in adolescents from three eastern Chinese provinces. Environ Health Prev Med [serial online]. 2018 [cited 2020 Oct 1]; 23(1):5. 10.1186/s12199-018-0695-2 PMC5803911 29415649

[30] Hansson M, Chotai J, Nordstöm A, Bodlund O. Comparison of two self-rating scales to detect depression: HADS and PHQ-9. Br J Gen Pract. 2009;59(566): 650–654. 10.3399/bjgp09X454070 PMC2734374 19761655

[31] Ceci SJ, Bruck M. Suggestibility of the child witness: A historical review and synthesis. Psychol Bull. 1993;113(3):403–439. 10.1037/0033-2909.113.3.403 8316609

